# Immunoproteomics reveal increased serum IgG3/5 binding to *Dermatophagoides* and yeast protein antigens in severe equine asthma in a preliminary study

**DOI:** 10.3389/fimmu.2023.1293684

**Published:** 2023-12-15

**Authors:** Christiane L. Schnabel, Maria-Christin Jentsch, Sabrina Lübke, Sarah Kaiser-Thom, Vinzenz Gerber, Susanne Vrtala, Huey-Jy Huang, Claudio Rhyner, Bettina Wagner, Ralf Hoffmann, Daniela Volke

**Affiliations:** ^1^ Institute of Immunology, Faculty of Veterinary Medicine, and Center for Biotechnology and Biomedicine, Leipzig University, Leipzig, Germany; ^2^ Swiss Institute of Equine Medicine (ISME), Department of Clinical Veterinary Medicine, Vetsuisse Faculty, University of Bern, Bern, Switzerland; ^3^ Division of Immunopathology, Department of Pathophysiology and Allergy Research, Center for Pathophysiology, Infectiology and Immunology, Medical University of Vienna, Vienna, Austria; ^4^ Christine Kühne Center for Allergy, Research, and Education (CK-CARE), Davos, Switzerland; ^5^ Swiss Institute of Allergy and Asthma Research (SIAF), Davos, Switzerland; ^6^ Department of Population Medicine and Diagnostic Sciences, College of Veterinary Medicine, Cornell University, Ithaca, NY, United States; ^7^ Institute of Bioanalytical Chemistry, Faculty of Chemistry and Mineralogy and Center for Biotechnology and Biomedicine, Leipzig University, Leipzig, Germany

**Keywords:** immunoglobulin isotypes, proteomics, 2D Western blot, LC-MS, asthma, COPD, RAO, heaves

## Abstract

**Introduction:**

Severe equine asthma (SEA) is a common, chronic respiratory disease of horses characterized by hyperreactivity to hay dust which has many similarities to severe neutrophilic asthma in humans. SEA-provoking antigens have not been comprehensively characterized, but molds and mites have been suggested as relevant sources. Here, we identified relevant antigen candidates using immunoproteomics with IgG isotype-binding analyses.

**Methods:**

Proteins from *Dermatophagoides pteronyssinus* (*Der p*) were separated by two-dimensional gel electrophoresis followed by immunoblotting (2D immunoblots) resulting in a characteristic pattern of 440 spots. After serum incubation, antibody (Ig)-binding of all Ig (Pan-Ig) and IgG isotypes (type-2-associated IgG3/5, type-1-associated IgG4/7) was quantified per each spot and compared between asthmatic and healthy horses’ sera (n=5 per group).

**Results:**

Ig binding differences were detected in 30 spots. Pan-Ig binding was higher with asthmatics compared to healthy horses’ sera on four spots, and IgG3/5 binding was higher on 18 spots. Small IgG4/7 binding differences were detected on 10 spots with higher binding with asthmatics’ sera on four but higher binding with healthy horses’ sera on six spots. Proteins from the spots with group differences including mite and yeast proteins were identified by liquid chromatography mass spectrometry. The latter likely originated from the feeding substrate of the *Der p* culture. Prioritized antigen candidates amongst the proteins identified were Der p 1, Der p 11, group 15 allergens, myosin heavy chain, and uncharacterized *Der p* proteins. Additionally, yeast enolases, alcohol dehydrogenase (ADH), phosphoglycerate kinase (PGK), glyceraldehyde-3-phosphate dehydrogenase, and heat shock proteins were prioritized. Eleven antigen candidates were tested for confirmation by ELISAs using the respective proteins separately. Differences in asthmatics vs. healthy horses’ serum Ig binding to Der p 1, Der p 18, and three yeast enzymes (enolase, ADH, and PGK) confirmed these as promising antigens of immune responses in SEA.

**Discussion:**

Antigens with relevance in SEA were newly identified by immunoproteomics, and yeast antigens were considered for SEA for the first time. Serum IgG3/5 binding to relevant antigens was increased in SEA and is a novel feature that points to increased type-2 responses in SEA but requires confirmation of the corresponding cellular responses.

## 1 Introduction

Severe equine asthma (SEA) is a common, chronic disease in adult horses with typical clinical signs like poor performance, mucus nasal discharge, coughing, and dyspnea (1, 2). It is caused by hyperreactivity of affected horses’ lower airways to hay dust exposure resulting in bronchospasm, mucus hypersecretion, and neutrophilic inflammation comparable to severe, neutrophilic asthma in humans (1, 2). To date, it is unclear if the pathogenesis of SEA is that of a typical allergy (Type I hypersensitivity, mediated by IgE). Nevertheless, the involvement of dysregulated adaptive immunity and a relevant contribution of antibodies (immunoglobulins, Ig) is likely (2). Molds as antigen sources have been studied widely, but the contribution of other hay components and contaminants like storage mites has been proposed (1, 2). Many analyses of allergen-specific IgE in equine asthma have been conducted but yielded conflicting results. While several studies found increased IgE against mold preparations (3, 4), *Aspergillus* (*Asp*) allergens (3, 5, 6), mite preparations (7, 8), and single mite allergens (3, 5) in asthmatic horses compared to healthy horses, others did not find significant differences in serological tests (9–12). Few studies analyzed IgG (6, 13, 14) and found increased Asp f 7- or Asp f 8-specific IgG in serum (6, 13) or *Asp* extract-binding IgG in BALF (15). Other than these, IgG analyses concerning SEA are sparse even though they could be informative: i) allergen-specific IgG isotypes are are usually induced in addition to IgE (16–18), and ii) IgG-dependent mechanisms like type III hypersensitivities and involvement of immune complex formation have been suggested as a pathological mechanism of equine asthma (2, 19, 20). Similarly, mouse models of human asthma indicated such mechanisms and increased allergen-specific IgG was associated with asthma in humans (21).

Nevertheless, the causative agents or provoking molecules in hay dust that cause SEA have not been defined. The contribution of bacteria, molds, plants, and arthropods, particularly storage mites, have been assumed based on provocation tests, observations, and serological comparisons (1, 2, 5, 8, 22). However, allergen testing *in vivo* and serological tests that rely on specific antibody binding depend on the correct choice of relevant antigens for investigation. This means that if the relevant antigens are not tested or are under-represented in a protein mixture used, like extracts, the test results will likely be inconclusive as group differences get blurred by non-specific effects (23). For the equine allergy *Culicoides* hypersensitivity, the use of the relevant allergens in pure form yields far more specific and sensitive results in serologic testing than the use of *Culicoides* extracts (24). For equine asthma, this is also indicated based on recent reports of allergen-specific IgE using a microarray of pure and mixed proteins (5). The allergens yielding the best accuracy in this assay were mainly pure allergen proteins (5).

However, disease-relevant antigens of SEA have not been identified comprehensively. Here, we employed a bottom-up approach to analyze the mite *Dermatophagoides pteronyssinus* (*Der p*) as a proxy for storage mites by immunoproteomics. Spatial separation of the different proteins by two-dimensional (2D) immunoblots enables the analysis of Ig binding to smaller groups of the proteins in single spots. As the relevance of IgE in SEA is unclear, we first aimed to identify antigens with immunogenicity that resulted in Ig production of any isotype but differed regarding the response provoked in asthmatic (SEA) vs. healthy horses. Since IgG isotypes comprise the majority of serum Ig, these were in focus. Furthermore, even in allergic diseases like *Culicoides* hypersensitivity, allergen-specific IgG, namely, equine IgG3 and IgG5 produced alongside IgE, can indicate sensitization and may contribute mechanistically (17). These IgG3/5 isotypes are preferentially provoked in a Th2-biased context, while Th1-biased immune responses of horses are associated with IgG4/7 isotypes in serum (25, 26). Therefore, we analyzed the binding of these two opposing IgG isotypes in serum separately to identify antigen candidates with relevance for SEA that provoke different Ig responses in asthmatic horses compared to healthy horses.

## 2 Materials and methods

### 2.1 Protein separation by two-dimensional electrophoresis and immunoblotting

Proteins were extracted from *Dermatophagoides pteronyssinus* (*Der p*) whole culture powder (Citeq, Groningen, Netherlands) by sonication (Branson 450 Digital Sonifier, 10 x 10 sec, max 35% amplitude, 20s breaks between pulses) in a pre-rehydration buffer (PrRhBu, 7 M urea, 2 M thiourea, 4% CHAPS; Carl Roth, Karlsruhe, Germany, 1 mL per 5 mg powder) on ice. The resulting suspension was cleared from insoluble debris by centrifugation. Total protein (*Der p* TP) was precipitated with 4-fold excess (v/v) of acetone at -20°C; the pellet dried in a vacuum centrifuge at room temperature (rt) and was then stored at -80°C. *Der p* TP was dissolved in PrRhBu and the protein concentration was determined by the Bradford method (RotiQuant, Carl Roth).

For isoelectric focusing (IEF), *Der p* TP (100 µg per strip) in PrRhBu containing 50 mM Dithiothreitol (DTT, Sigma/Merck KGaA, Darmstadt, Germany), Ampholyte (1x, 100x BioLyte^®^ 3/10 Ampholyte, BioRad, Feldkirchen, Germany), and 0.001% bromophenol blue (w/v Sigma) was applied to an IPG BlueStrip (pH 3-10 NL 7cm, Serva, Heidelberg, Germany) and allowed to hydrate for 6 h at rt. IEF was performed in a Protean IEF System (BioRad) covered with mineral oil (BioRad) with the following steps: active rehydration, 50 V, 6 h; conditioning step, 150 V rapid ramp, 1 h; conditioning step, 300 V rapid ramp, 1 h, voltage Ramping, 1000 V linear ramp, 1 h, voltage ramping, 3000 V linear ramp, 2 h; final focusing, 3000 V rapid ramp, 2 h; hold, 500 V, up to 12 h (27). The strips were then equilibrated with 0.19 M DTT for 20 min, followed by 0.43 M iodoacetamide (Sigma) for 25 min (each in 0.05 M TrisHCl, pH 8.8, 6.3 M urea, 20% (v/v) glycerol, 2% (w/v) sodium dodecyl sulfate (SDS)), at rt with gentle agitation.

Polyacrylamide (PA) gels were prepared in a Mini Protean Gel casting system (BioRad) with two equal parts of a 14% PA separation (with 10% glycerol) and a 10% PA spacer gel in 1 M Tris, 0.1% (w/v) SDS, pH 8.45 (28), with 0.5% (v/v) 2,2,2-Thrichloroethanol (TCE) and polymerization induced with 0.03% (w/v) APS and 0.003% TEMED (all from Sigma) (27). The focused, equilibrated IPG strips were attached on top of the PA-gels with warm 0.5% agarose (Biozym, Hessisch Oldendorf, Germany) in cathode buffer, and gel electrophoresis (GE) was run between the cathode buffer (0.1 M Tris, 0.1 M Tricine (N-[Tris(hydroxymethyl)methyl]-glycine), 0.1% (w/v) SDS, pH 8.25) and anode buffer (0.2 M Tris pH 8.9) (28).

TCE reaction with the separated proteins was activated (29) by UV exposure of each gel for 1 minute, and the protein pattern according to the resulting tryptophan fluorescence (TF) was recorded as ‘StainFree’(SF) (**Table 1**) on a BioRad ChemiDoc MP equipped with ImageLab software (BioRad). The gels were then blotted on nitrocellulose membranes (0.2 µm, CarlRoth) in a tank blot procedure (19.8 mM Tris, 0.15 M glycine, 20% v/v ethanol all Carl Roth) at 300 mA for 30 min. After the transfer, control images of SF, Cy3, and Cy5 fluorescence of the membranes were acquired (ChemiDoc MP; **Table 1** membranes). All the following membrane incubation steps were performed protected from light with gentle agitation.

**Table 1:** Fluorescent protein and immunodetection.

Target	Fluorescence channel^1^	Exposure time (seconds)	Detection antibody	Secondary antibody/fluorochrome	Reference, source
**Protein**	Stain Free	*8 (gels)* 2 (membranes)	/	(Tryptophan fluorescence, TF)^2^	(29)
**IgG3/5**	Cy5	2	Anti-IgG3/5 clone 586	Alexa Fluor^®^ 647 AffiniPure Goat Anti-Mouse IgG (H+L) [Anti-mouse-A647]	(30) JIR, 115-605-146
**IgG4/7** ^3^	Cy5	2	Anti-IgG4/7 clone CVS39	[Anti-mouse-A647]	(30, 31) JIR, 115-605-146
**Pan-Ig** ^3^	Cy3	5	Cy™3 AffiniPure Goat Anti-Horse IgG (H+L)	[Anti-horse-Cy3]	JIR 108-165-003

^1^ on ChemiDoc MP (BioRad); ^2^ TF is evoked by UV activation of TCE and its modification of tryptophan residues to visualize protein; ^3^ multiplexed detections on the same membrane.

The membranes were blocked in 1x Blue block PF (Serva) and then incubated with horse serum (**Table 2**) diluted 1:250 in Blue block PF (Serva) at 4°C overnight. After washing in TBST (20 mM Tris, 150 mM NaCl, 0.5% v/v Tween20, all Sigma), the membranes were incubated with detection antibodies anti-IgG3/5 or anti-IgG4/7 (**Table 1**) diluted in Blue block PF at rt. After washing in TBST, the membranes were incubated with A647 fluorochrome-conjugated secondary antibodies (Anti-mouse-A647, and/or Anti-horse-Cy3, Jackson ImmunoResearch (JIR), Dianova, Hamburg, Germany, **Table 1**) in Blue block PF at rt. After washing in TBST and water, the membranes were imaged (‘detection’ ChemiDoc MP), and SF, Cy3, and Cy5 fluorescence were recorded (**Table 1**).

**Table 2:** Serum donor horse characteristics.

Barn	Group	Sex	Breed	Age (years)	Clinic^1^	HOARSI^2^
**A**	Asthmatic	Mare	Appaloosa-Quarter-Mix	20	3	4
**A**	Healthy	Gelding	Franches-Montagnes	18	0	1
**B**	Asthmatic	Gelding	Pony-Mix	20	3	4
**B**	Healthy	Mare	Swiss Warmblood	13	0	1
**C**	Asthmatic	Gelding	Pony-Mix	28	3	4
**C**	Healthy	Gelding	Arabian	25	0	1
**D**	Asthmatic	Mare	Haflinger	20	3	4
**D**	Healthy	Mare	Haflinger	8	0	1
**E**	Asthmatic	Gelding	Criollo	18	3	4
**E**	Healthy	Gelding	Criollo	18	0	1

^1^ clinical score evaluating the severity of mucous nasal discharge, cough, and dyspnea at rest (0 healthy, 3 severe symptoms); ^2^ Horse Owner Assessed Respiratory Signs Index (32).

### 2.2 Sera

Equine sera were acquired from severely asthmatic horses and healthy controls (**Table 2**), which were included in a previous study (33) under the animal experiment permission number BE110/16+ and were stored frozen until use in the experiments. Sera from healthy controls or asthmatic horses were selected after classification based on the horses’ histories (HOARSI 1 or 4, respectively) (32) and the absence or presence of clinical signs of asthma upon physical examination at the time of blood sampling by the conducting veterinarian, summarized by a clinical score (0 or 3, respectively) (33). Each healthy and asthmatic horse pair was matched by their environment (barn) and each was sampled on the same day between June and October 2017. The horses’ endoparasite burden was considered low according to counts of 0 – 150 eggs per gram of feces (McMaster method), with a median of 0 in both groups.

### 2.3 Immunoproteomics data evaluation

The raw data of the 2D immunoblot images were exported via ImageLab software (BioRad) and then imported into Delta 2D software (DECODON, Greifswald, Germany) as a classic project standardized by fluorescence volumes. Hierarchical warping was set up as follows: all gels (protein spot pattern) were warped exact and a fused image was generated. All membranes (protein spot pattern after transfer) were warped exact to their corresponding gel, and the membranes at detection were warped to those after transfer (exact). The images from the detection channels (Cy3 for Pan-Ig, Cy5 for IgG3/5 or IgG4/7) were warped identical to the respective protein pattern image of that membrane (TF at detection).

On the fused image, spots were detected automatically and manually corrected to exclude false detections at the edges as they were in the very acidic or alkaline regions with insufficient separation or were not visually identified on all gel images, resulting in 440 spots (**Supplementary Figures 1A, B**). To ensure consistent transfer of the protein, the membranes after transfer were block-randomized into eight groups, and for each spot, TF volumes were compared between each pair of groups: ratios of the standard deviation (RSD) were analyzed via the statistics tool in Delta 2D using medians for the calculation. Spots with RSD >2, indicative of ‘regulation’, in more than one comparison of the groups were considered inconsistent and excluded from further analyses for immunoproteomics (15 spots). Thereafter, 425 spots remained for analysis of their immunodetection (serum Ig binding; example in **Supplementary Figure 2**). The clustering of the immunodetection membranes was analyzed using the statistics tool and hierarchical clustering in Delta 2D (**Supplementary Figure 3**). Integrated grey volumes of each spot (without background) were exported from Delta 2D for each fluorescence channel and were log-transformed to achieve a normal distribution.

### 2.4 Statistical analysis and ranking of spots of interest

Statistical analyses were performed with GraphPad Prism software (GraphPad, L Jolla, CA, USA) volume 8 or higher. For each spot and isotype analyzed, the log-transformed fluorescence intensity volumes, as a measure of serum Ig binding, were compared between sera from asthmatic vs. healthy horses by repeated measures ANOVA and Benjamini, Krieger, and Yekutieli testing using an FDR of 5% (q-values), correcting for multiple comparisons between all spots. To also analyze each spot independently, comparisons with Fisher’s LSD test with alpha 1% were used for each isotype (p-values). For spots prioritized according to these comparisons, serum Ig binding was again compared between healthy and asthmatic horses’ sera with consideration of multiple comparisons between Pan-Ig and the isotypes IgG3/5 and IgG4/7, using Sidak’s tests with alpha of 5%.

Spots were prioritized to likely contain asthma-relevant antigen candidates if group differences of serum Ig binding between asthmatic and healthy horses’ sera were indicated by discoveries in the ANOVA with Benjamini, Krieger, and Yekutieli test’s q<0.05 comparing Pan-Ig, IgG3/5, or IgG4/7 binding group differences of all spots, or Benjamini, Krieger, and Yekutieli test’s q <0.1 and Fisher’s LSD test of p< 0.01, or Fisher’s LSD test of p< 0.01 in several isotypes and confirmation in the Sidak’s test (p<0.05) comparing Pan-Ig, IgG3/5, and IgG4/7 binding group differences per spot (**Supplementary Figure 4**).

Additionally, for Pan-Ig and the two isotypes, control spots were selected, which had strong Ig binding (log median fluorescent volume >1) and similar Ig binding with healthy and asthmatic horses’ sera (p>0.1).

### 2.5 Liquid chromatography-mass spectrometry

To identify the proteins contained, the prioritized spots with group differences and control spots were subjected to LC-MS in two independent experiments. IEF and PAGE of *Der p* protein were performed as described for the 2D immunoblots above but without TCE. After IEF and PAGE, the gels were stained with Coomassie brilliant blue (G-250) and fixed (50% (v/v) Methanol and 10% (v/v) acetic acid). The selected spots were cut out from these gels using a BioRad Exquest spot cutter (BioRad) (**Supplementary Figure 1C**). In comparison, 100 ng of the whole *Der p* protein digest obtained by FASP (34) was also subjected to LC-MS using a 90 min gradient instead of 40 min.

Protein digest was performed as previously described (35). Briefly, excised gel spots were transferred into Mt-plate (ThermoFisher). Gel pieces were washed three times (5 min, 100 µL 30% (v/v) acetonitrile in 50 mM ammonium bicarbonate), dehydrated with acetonitrile (5 min, 100 µL), and rehydrated with a mixture of 2 µL trypsin solution (Serva, 50 ng/µL in 3 mM aqueous ammonium bicarbonate) and 18 µL of 3 mM aqueous ammonium bicarbonate. After incubation (37°C, 4 h), supernatants were transferred to new 0.5 mL reaction tubes. The remaining gel pieces were washed once with 60% (v/v) aqueous acetonitrile containing 0.1% (v/v) formic acid and acetonitrile (20 µL per tube, RT, 5 min). Supernatants were transferred to the corresponding reaction tube and dried (60°C, 1 h) in a vacuum concentrator 5301 (Eppendorf, Hamburg, Germany). The dried digests were dissolved in a mixture of 1.5 µL of acetonitrile containing 0.1% (v/v) formic acid (eluent B) and 48.5 µL of 0.1% aqueous formic acid (eluent A) and separated on a nanoACQUITY Ultra Performance LC™ (Waters Corp., Manchester, UK) system coupled online to a Q-TOF SYNAPT G2-Si instrument (Waters Corp., UK). Peptides were trapped on a nanoACQUITY Symmetry C18-column, internal diameter (ID) of 180 µm, length of 2 cm, particle diameter of 5 µm, and flow rate of 5 µL/min (3% eluent B, 6 min) on a C18-BEH 130 column (ID of 75 µm, length of 10 cm, and particle diameter of 1.7 µm; 35°C) at a flow rate of 0.3 µL/min using linear gradient from 3% to 40% eluent B in 18.5 min. The nanoESI source was equipped with a PicoTip Emmitter (New Objective, Littleton, USA) at a spray voltage of 3 kV, with a sampling cone of 30 V, source offset of 80 V, source temperature of 100°C, cone gas flow of 20 L/h, and nanoflow gas pressure of 0.2 bar. Mass spectra were recorded in positive ion mode using a high-definition data-dependent acquisition approach (HD-DDA) for the top six ions.

LC-MS/MS raw files were processed with the Mascot search engine (Version 2.7.0; Matrix Science Ldt., Waters, UK) using the following parameters: UniProtKB Database with entries from *Dermatophagoides* and yeast with 38215 entries downloaded on 23.12.21; enzyme trypsin and 2 miss cleavage sides as fixed modification cysteine carbamidomethylation (+57.022 Da) and as variable modification methionine oxidation (+15.9949 Da), 20 ppm peptide tolerance, and 0.08 Da fragment tolerance. Proteins identified by at least three peptides and a peptide score ≥ 50 were considered confident.

In the resulting data, those from vertebrate species were excluded and the remaining proteins consolidated if identical peptides were attributed to several entries. Then, the proteins identified were prioritized according to the frequency of their overall appearance in all spots if they were a previously described allergen, they were confirmed in the duplicate LC-MS analysis, and they were not detected in the control spots.

### 2.6 Confirmation of selected proteins by ELISA

Selected antigens available as pure proteins were used in ELISAs (**Table 3**) as previously described for Tetanus toxoid as a model antigen (40). Briefly, proteins (4 µg/mL) in 0.05 M sodium carbonate buffer pH 9.6 (Sigma) were coated on MaxiSorp plates (Flat Bottom, Nunc, Thermofisher) at 4 °C overnight and the plates were blocked with 0.1% (w/v) gelatin and 0.5% bovine serum albumin (BSA) in PBS for 1 h at rt. All following steps were performed at rt and separated by washes with PBST (PBS, 0.05% Tween-20). Sera were diluted in serum diluent (PBS, 1% gelatin and 2.5% bovine serum albumin, 0.05% Tween-20; **Table 2**) according to preliminary experiments to be in their linear titration range (**Table 3**) and incubated for 2 h at rt, followed by polyclonal Peroxidase Goat-anti-horse-Ig (H+L) (#108-035-003, JIR), or monoclonal detection antibodies (Anti-IgG3/5 or Anti-IgG4/7, **Table 1**) and Peroxidase Goat-anti-Mouse-IgG(H+L) (#115-035-146, JIR) in PBST, and TMB substrate solution (medac GmbH, Wedel, Germany) stopped by sulfuric acid (Roth). Resulting optical densities at 450 nm were recorded with a SpectraMax 340 instrument and SoftMax Pro software (Molecular Devices, ThermoFisher).

**Table 3:** Proteins used in ELISAs for antigen-specific Ig quantification.

Proteins	Origin species	Source	Catalog number or reference	Comments (allergen information)
Arthropod proteins				
Der p 1 (recombinant ^a^, purified)	*Dermato-phagoides pteronyssinus (Der p)*	Claudio Rhyner, SIAF	(5, 23)	Cysteine protease
Der p 7 (recombinant ^a^, purified)	*Der p*	Prof. Vrtala, Vienna	(36)	Lipid-binding protein
Der p 11 (recombinant ^a^, purified)	*Der p*	Prof. Vrtala, Vienna	(37)	Paramyosin
Der p 15 (recombinant ^a^, purified)	*Der p*	Prof. Vrtala, Vienna	(38)	Chitinase
Der p 18 (recombinant ^a^, purified)	*Der p*	Prof. Vrtala, Vienna	(39)	Chitinase-like protein
Glyceraldehyde-3-phosphate dehydrogenase 1	*Drosophila melanogaster (D.m.)*	Cusabio technology LLC, Houston, TX, USA	CSB-EP362145DLU-20	(GAPDH), not described as allergen
Fungal proteins				
Enolase	*Saccharomyces cerevisiae (S.c.)*	Sigma	E6126	Compare Cand a Enolase
Alcohol dehydrogenase (ADH)	*S.c.*	Merck	A7011	not described as allergen
3-Phosphoglycerol-phosphokinase (3-PGK)	*S.c*	Merck	P7634	not described as allergen
Triosephosphate isomerase (TPI)	*S.c.*	Merck	T2507	not described as allergen
Actin (ACT1) (recombinant ^a^, purified)	*S.c.*	Cusabio	CSB-EP001205SVG	not described as allergen
Actin (ACT1) (recombinant ^a^, purified)	*Absidia glauca (A.g.)*	Cusabio	CSB-EP320814AAD	not described as allergen

a expressed in *E. coli*.

### 2.7 Total serum Ig isotype quantification

The total concentrations of Ig isotypes in the sera were quantified by bead-based assays at the Wagner laboratory, College of Veterinary Medicine, Cornell University, Ithaca, NY, USA, as previously described (30, 41). IgM, IgG1, IgG3/5, IgG4/7, and IgG6 were quantified in a multiplexed assay with sera diluted 1:50,000. IgE concentrations were determined in a singleplex assay with sera diluted 1:10.

Total Ig isotype concentrations (in median fluorescence intensities, MFI) and blank-reduced optical densities (OD) from ELISAs were compared between healthy and asthmatic horses’ sera using Wilcoxon Signed-Rank tests.

## 3 Results

### 3.1 2D Immunoblots reveal IgG3/5 binding differences on distinct spots

**Figure 1:**
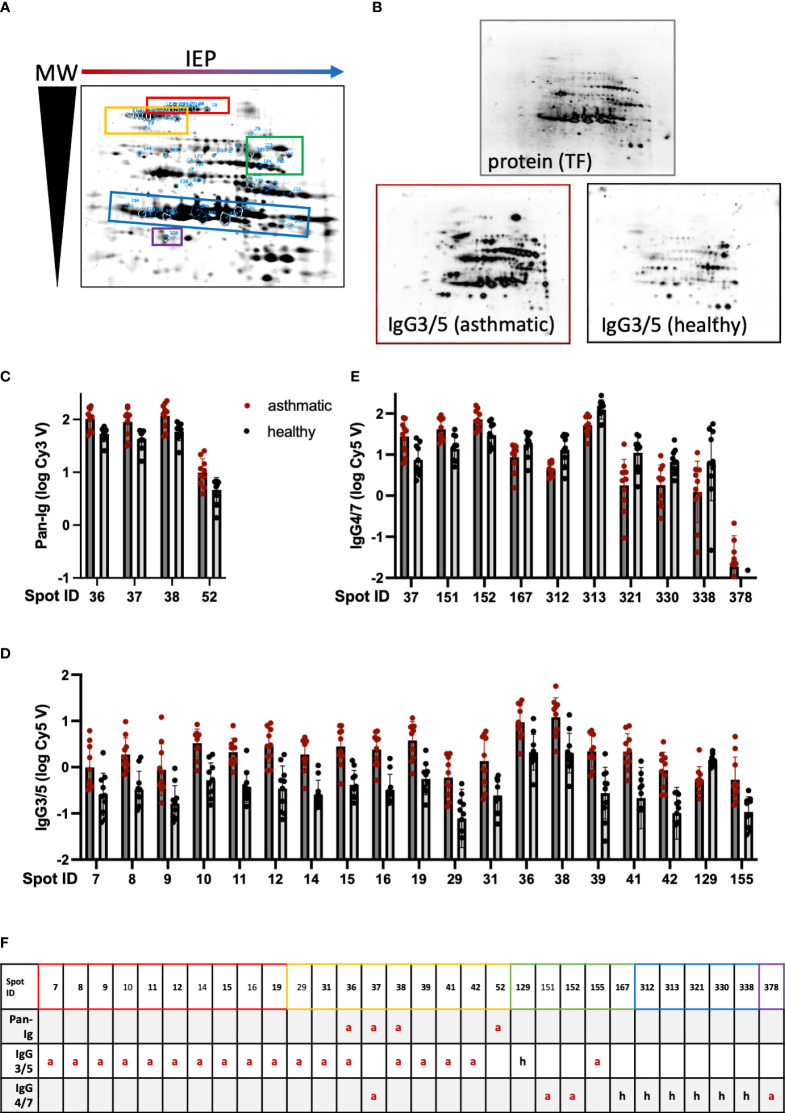
Serum Ig binding differences detected on 2D immunoblots indicate distinct spots to contain antigens relevant in Severe Equine Asthma (SEA). *Dermatophagoides pteronyssinus* (whole culture) protein was separated by isoelectric point (IEP) and molecular weight (MW) on polyacrylamide gels resulting in a characteristic pattern of 440 spots. **(A)** A fused image of the protein spot pattern on the gels is depicted. **(B)** The protein was blotted on nitrocellulose membranes and these were incubated with serum of asthmatic or healthy horses (duplicates of n=5 per group) followed by detection antibodies to quantify serum Ig binding by fluorescence. Protein is visualized by tryptophan fluorescence (TF). A representative visualized example of IgG3/5 detection in a serum pair (asthmatic and healthy horse from the same environment) is depicted. **(C)** Pan-Ig, **(D)** IgG3/5, and **(E)** IgG4/7 binding (logarithmized fluorescence intensity volumes) to spots with significant differences between asthmatic (dark grey bars) and healthy (light grey bars) horses’ sera are plotted. Bars indicate mean and SD. Group differences in Pan-Ig and IgG3/5-binding were indicated by Benjamini, Krieger, and Yekutieli’s *post-hoc* test (q<0.05). Group differences in IgG4/7-binding were indicated by Benjamini, Krieger, and Yekutieli’s *post-hoc* test (q<0.01) for spot 167, and by Fisher’s LSD test (p<0.01) for the other spots. **(F)** Group differences between asthmatic and healthy horses’ sera are indicated for each prioritized spot and Ig isotype (or Pan-Ig) (red a: higher Ig binding with asthmatics; black h: higher Ig binding with healthy horses’ sera). Spots successfully cut and analyzed by LC-MS are indicated by bold spot IDs **(F)** and are highlighted on the gel image **(A)** by blue spot boundaries and spot ID annotation as well as control spots. Spot groups are indicated by colored boxes **(A, F)**. Spots with significant differences in Ig binding between asthmatic and healthy horses’ sera were selected for further analysis. Double spot boundaries indicate spots prioritized.

Separation of the *Der p* proteins resulted in 440 distinct spots (**Figure 1A**) of which 425 were evenly transferred onto nitrocellulose membranes and considered in the analysis of serum Ig binding per spot and isotype (**Figure 1B**). Hierarchical clustering including all spots per Pan-Ig/isotype analyzed showed clustering of duplicates (**Supplementary Figure 3**). However, Ig binding patterns over all spots were not strictly separated by asthma or a healthy status, and all three Ig binding patterns were independent of the environmental matching of the horses (barn **Supplementary Figure 3**).

Differences in serum Ig binding between asthmatic and healthy horses’ sera were analyzed per spot and isotype. Pan-Ig, IgG3/5, or IgG4/7 binding differences were observed for a total of 30 spots (**Figures 1C–F**). The binding of all serum Ig (Pan-Ig) detected by a polyclonal antibody against equine Ig heavy and light chains (**Table 1**) was only different between the groups’ sera for four spots. These (IDs 36, 37, 38, and 52) were all in a group in the high MW acidic part (upper left) of the spot pattern (**Figure 1A**, yellow), and each spot yielded higher binding by asthmatic than healthy horses’ serum Ig (p<0.05 in Benjamini, Krieger, and Yekutieli *post-hoc* tests; **Figures 1C, F**).

Similarly, IgG3/5 binding was usually higher with asthmatics’ sera than with healthy horses’ sera and the group differences were statistically significant for 19 spots (**Figure 1D**) in several groups on the higher MW part (upper half) of the spot pattern (IDs 7–155; **Figure 1A**, red and yellow). IgG3/5 binding was only higher with healthy horses’ sera compared to asthmatic horses’ sera for spot 129, which is in the medium-high MW area and alkaline part of the gel/membrane (middle right; **Figure 1A**, green).

Despite overall high IgG4/7 signals (high fluorescence intensities; **Supplementary Figure 4**), binding differences of IgG4/7 between the groups were less robust than those of Pan-Ig or IgG3/5. IgG4/7 only yielded a group difference of asthmatic vs. healthy horses’ sera for spot 167 with Benjamini, Krieger, and Yekutieli *post-hoc* tests (p<0.1), while for 10 more, spot differences were detected according to Fisher’s LSD tests (without consideration of multiple comparisons between all spots, p<0.05; **Figure 1E**). Spots in the groups of previously mentioned areas (IDs 37, 151, and 152, all high MW) and spot 378 (lower left; **Figure 1A**, purple) also yielded higher IgG4/7 binding with asthmatic horses’ sera than with healthy horses’ sera (**Figures 1E, F**). However, for spot 167 and most spots in medium low MW areas (IDs 312 – 338, lower half of the spot pattern; **Figure 1A**, blue), lower IgG4/7 binding with asthmatic horses’ sera was observed compared to that with healthy horses’ sera (**Figures 1E, F**).

Three spots yielded group differences in Ig binding in Pan-Ig *and* IgG3/5 (IDs 36 and 38) or Pan-Ig *and* IgG4/7 (ID 37), while the Pan-Ig binding difference for spot 52 was not reflected in IgG3/5 or IgG4/7 in comparison to all spots (**Figure 1F**; **Table 4**). In the isotype comparison of spot 52 alone, however, increased IgG3/5 binding of asthmatic compared to healthy horses’ sera was also indicated (**Supplementary Figure 4**). Overall, increased IgG3/5 binding of asthmatic horses’ sera compared to healthy horses’ sera was the most common pattern observed with the most robust differences between the groups (**Figures 1D, F**; **Supplementary Figure 4**).

**Table 4:** Antigen candidates identified by immunoproteomics in spots with Ig binding differences of asthmatic and healthy horses’ sera.

Protein description ^a^	LC-MS^b^	Protein group/function ^c^	Allergen description ^d^	*ELISA ^e^*	Spots of inte-rest ^f^	control ^g^	Der p TP ^h^	Spot IDs	7	8	9	1 1	1 2	1 5	1 9	3 1	3 6	3 7	3 8	3 9	4 1	4 2	5 2	1 2 9	1 5 2	1 5 5	1 6 7	3 1 2	3 1 3	3 2 1	3 3 0	3 3 8	3 7 8
							Pan-Ig binding ^i^										a	a	a				a										
							IgG3/5 binding ^i^		a	a	a	a	a	a	a	a	a		a	a	a	a		h		a							
							IgG4/7 binding ^i^											a							a		h	h	h	h	h	h	a
ARTHROPOD PROTEINS																																	
**Der p 1 allergen (Fragment);** Major fecal allergen Der p I (Fragments); Der p 1 variant (Fragment)	1	EN cysteine protease	Group 01ft	Pan-Ig, IgG4/7	3	***n***	x																						x		x		x
**Cysteine proteinase-1 preproenzyme (Fragment)**	1	EN cysteine protease	comp. Der p 1		5	**n**	x																				x	x	x		x	x	
**Der f 35-like allergen (Fragment);** mite group 2 allergen Pso o 2-like	0	MD-2-like protein; Der f 35-like, NPC2 family	Group 02		1	**n**	x								x																		
**Mite allergen Der p 3;** Der p 3 allergen; Der f 3 allergen	0	EN trypsin	Group 03		2	**n**	x																								x	x	
**Mite allergen Der f 6-like;** Der f 6 allergen	1	EN chymotrypsin	Group 06		2	**n**	x																						x				x
**Mite allergen Der p 7;** Der f 7 allergen	0	Bactericidal permeability-increasing like protein	Group 07	ns	1	**n**	x																					x					
**98kDa HDM allergen;** HDM allergen; Der f 11 allergen	1	Paramyosin	Group 11	ns	14	**n**	x		x	x	x	x	x	x	x	x	x	x	x	x											x	x	
**Group 15 allergen protein short isoform;** Group 15 allergen protein	0	EN chitinase	Group 15		13	**n**	x		x	x	x	x	x	x	x	x	x	x	x	x		x											
**Chitinase-3-like protein 1**	1	EN chitinase	Group 15		14	**n**	x		x	x	x	x	x	x	x	x	x	x	x	x	x	x											
Chitinase-like mite allergen Der f 18.0101	0	EN chitinase-like	Group 18	IgG3/5	0	**n**	x																										
**Der f 16 allergen**; Der f 16-like allergen (Fragment)	0	Gelsolin/Villin	Group 16		2	**n**	x			x															x								
**Der f 28-like allergen (Fragment);** Der f 28 allergen	0	HSP	Group 28		1	**n**	x															x											
**Der p 36 allergen**	0	uncharac-terized	Group 36		1	**n**																										x	
**ATP synthase subunit beta**	1	EN ATP synthase	comp. Pla l		2	**n**	x																					x	x				
**Aldehyde dehydrogenase (NAD(+))**	1	EN dehydro-genase			1	**n**	x			x																							
**Cathepsin L1-like**	1	EN cystein proteinase			2	**n**	x																					x	x				
**Fumarate hydratase**	2	EN fumarate hydratase			1	**n**																				x							
Glyceraldehyde-3-phosphate dehydrogenase	0	EN dehydro-genase	*comp. yeast*	ns	1	**n**	x																							x			
**Pancreatic triacylglycerol lipase-like**	1	EN lipase			6	**n**	x					x						x		x	x	x	x										
**Serine hydroxymethyl-transferase**	1	EN transferase			1	**n**	x																			x							
**Heat shock protein 83**	1	HSP	*comp. yeast*		3	**n**						x		x						x													
**14-3-3 protein zeta isoform X2; X3**	1	14-3-3 protein (adapter)	*comp. yeast*		3	**n**	x								x													x	x				
**Actin, cytoplasmic A3a**; LOW QUALITY PROTEIN: actin-4-like	1	actin	*comp. yeast*		4	**n**	x												x								x	x	x				x
**Myosin heavy chain, muscle-like isoform X4**	1	myosin			15	**n**	x		x		x	x	x	x		x	x		x	x			x	x			x	x	x				x
Myosin heavy chain, muscle-like	0	myosin			6	**n**	x		x		x		x	x		x			x														
Keratin, type I cytoskeletal 14-like	0	keratin			3	**n**	x																			x	x	x					
uncharacterized protein LOC113793118	1	uncharac-terized			3	**n**	x																					x	x				x
**uncharacterized protein LOC113796964**	1	uncharac-terized			9	**n**	x					x	x	x		x		x		x	x	x	x										
**uncharacterized protein LOC113796965**	1	uncharac-terized			2	**n**	x					x								x													
uncharacterized protein LOC113797715	0	uncharac-terized			3	**n**	x																		x			x			x		
**uncharacterized protein LOC113799427**	1	uncharac-terized			14	**n**	x		x	x		x	x	x		x	x		x				x	x	x					x	x	x	
FUNGAL PROTEINS																																	
**Enolase 1;** Enolase; Enolase 2	1	EN enolase	Sac c Enolase; comp. Cand a Enolase; Alt a 6, Asp f 22	Pan-Ig (IgG3/5)	23	**n**	x		x	x	x	x	x	x	x	x	x	x	x	x	x	x		x	x	x	x	x	x	x		x	x
**Alcohol dehydrogenase 1;** 2	***1***	EN dehydro-genase	*comp. Cand a 1*	IgG4/7	17	y	x		x	x	x	x	x	x	x	x	x		x					x			x	x	x	x	x		x
**Phosphoglycerate kinase**	1	EN kinase	0	(Pan-Ig), IgG3/5	14	**n**	x		x	x	x	x	x	x	x		x								x		x	x		x	x		x
**Glyceraldehyde-3-phosphate dehydrogenase 1;** 2; 3	***1***	EN dehydro-genase	*comp. mite*	*(ns)*	10	**n**	x		x	x	x						x		x									x	x	x	x	x	
**Pyruvate kinase 1**	0	EN kinase			13	**n**	x		x	x	x	x		x	x		x		x	x						x				x	x	x	
**Triosephosphate isomerase**	1	EN Isomerase	*comp. Asp t 36*	(IgG3/5, IgG4/7)	1	**n**																											x
**Bifunctional purine biosynthetic protein ADE5,7**	1	EN ligase			1	**n**														x													
**Glycerol-1-phosphate phosphohydrolase 1;** 2	1	EN phospho-hydrolase			1	y	x																					x					
**Phosphomanno-mutase**	1	EN mutase			2	**n**	x																					x	x				
**Phosphoglycerate mutase 1**	1	EN mutase			2	**n**	x																								x	x	
**V-type proton ATPase subunit E**	1	EN ATPase			3	**n**	x																					x	x				x
**Heat shock protein SSA1; SSA2**	0	HSP	*comp. mite*		8	**n**	x					x						x		x	x	x		x				x					x
**Heat shock protein 26**	1	HSP	*comp. mite*		4	**n**	x																					x	x		x		x
**ATP-dependent molecular chaperone HSP82;** HSC82; Heat shock protein 90 homolog	1	HSP	*comp. mite*		6	**n**	x					x		x				x		x	x	x											
**Heat shock protein homolog SSE1**	1	HSP	*comp. mite*		2	**n**	x					x								x													
14-3-3 protein homolog	0	14-3-3 protein (adapter protein)	*comp. mite*		1	**n**	x																						x				
**Actin**	1	actin	*comp. mite*	(Pan-Ig)	3	**n**	x																					x	x				x

^a^ all hits for the same protein are listed; bold: prioritized if 2 out of these criteria met: in min. 5 spots, not in control spots, confirmed in duplicate LC-MS, described allergen; underlined: 3 or 4 criteria met, highest priority; **^b^** confirmed in duplicate 2D PAGE and LC-MS 2 exact; 1 isoforms or homologues; 0 only detected in one analysis; **^c^** EN enzyme, HSP heat shock protein; **^d^** allergen name/group; comp. comparable allergen, or class similar in yeast/mite; **^e^** isotype with difference; () trend in this isotype; grey homologue tested; **^f^** number of spots; **^g^** detected in control spots without group differences n no, y yes; **^h^** x detected; **^i^** Ig binding to these spots per isotype a higher in asthmatic, h higher in healthy.

### 3.2 Proteins identified as antigen candidates

All spots with differences in Ig binding between healthy and asthmatic horses’ sera were selected for further analysis by LC-MS (**Figure 1A**, double spot borders). In addition, six immunogenic control spots (IDs 131, 184, 255, 306, 318, and 325) with high Ig binding but without group differences were selected for comparison. Also, the total *Der p* protein (*Der p* TP) was analyzed by LC-MS (**Table 4**). All results can be accessed via https://panoramaweb.org/Der-p-in-SEA.url. Of the 30 spots of interest, 25 were successfully cut after two separations of 2D GE with Coomassie staining and subjected to LC-MS analysis (in duplicate; **Table 4** and **Supplementary Figure 1**). In the spots of interest, peptides of fungal proteins, particularly yeast proteins, were over-represented over those of arthropod (mite) proteins. This was not the case in the analysis of the whole *Der p* protein (*Der p* TP, *Dermatophagoides pteronyssinus* whole culture), which also yielded identifications of fungal proteins, although more mite proteins were identified in this mixture (**Table 4**).

Highly similar proteins, such as isoforms of the same allergen (e.g., Der p 1) or homologs like Der p 1 and Der f 1 of *Dermatophagoides farinae* were summarized for the final presentation (**Table 4**; **Supplementary Table 1**). Several proteins of similar classes of both arthropod and fungal origin were identified as antigen candidates, but they frequently had low protein identity (<70%) according to protein-protein BLAST analyses, and were listed separately as antigen candidates, such as the enzyme glyceraldehyde-3-phosphate dehydrogenase (GAPDH), heat shock proteins, 14-3-3 adapter proteins, and actin of arthropod and fungal origin (**Table 4**; **Supplementary Table 1**).

The arthropod proteins identified comprised several previously described *Dermatophagoides pteronyssinus* and closely related *Dermatophagoides farinae* allergens, structural proteins, and unknown proteins. The proteins in the spots of interest were usually also detected in the *Der p* TP but did not simply reflect its composition. For example, Der p 1 and Der p 2 were frequently identified in *Der p* TP, but only Der p 1 was repeatedly identified in the spots of interest.

The allergens Der p 1 (cysteine protease), Der p 11 (98kDa HDM allergen, paramyosin), Der p 15 (chitinase), and chitinase-3-like protein 1 (group 15 allergen) were identified with the highest certainty according to their frequency of peptide identification, with confirmation in duplicate LC-MS analyses and absence in control spots. Der p 1 was only detected in the low MW spots (IDs 313, 330, and 378; **Figures 1A, F**) matching its size of 25 kDa. Der p 11 and the group 15 allergens were detected in 14 spots in the upper MW range corresponding to their higher MW of 98 kDa and 60 kDa, respectively (**Figures 1A, F**; **Table 4**). Further mite proteins prioritized as antigen candidates were myosin heavy chain (muscle-like isoform X4) and two uncharacterized proteins (LOC113796964 and LOC113799427).

The fungal proteins from the spots of interest with Ig binding differences were abundantly detected in the spots of interest and contained many enzymes, such as yeast enolases (enolase 1 and enolase 2) in 23 spots of interest, alcohol dehydrogenase (ADH) in 17 spots (and control spots), phosphoglycerate kinase (PGK) in 14 spots, and glyceraldehyde-3-phosphate dehydrogenase (GAPDH) in 10 spots. Furthermore, heat shock proteins (HSP) were among the prioritized antigen candidates, particularly HSP82 detected in six spots in the high MW areas matching its MW of 82 kDa (**Figures 1A, F**; **Table 4**). Actin of arthropod origin was identified as an antigen candidate in four medium-low MW spots matching its MW of 42 kDa (**Figures 1A, F**; **Table 4**).

Overall, the immunoproteomics approach used here pointed to known mite allergens as antigen candidates, as well as new targets from mites, and particularly enzymes from yeasts.

### 3.3 Confirmation of antigens provoking Ig binding differences of asthmatic horses’ sera compared to healthy horses’ sera

**Figure 2:**
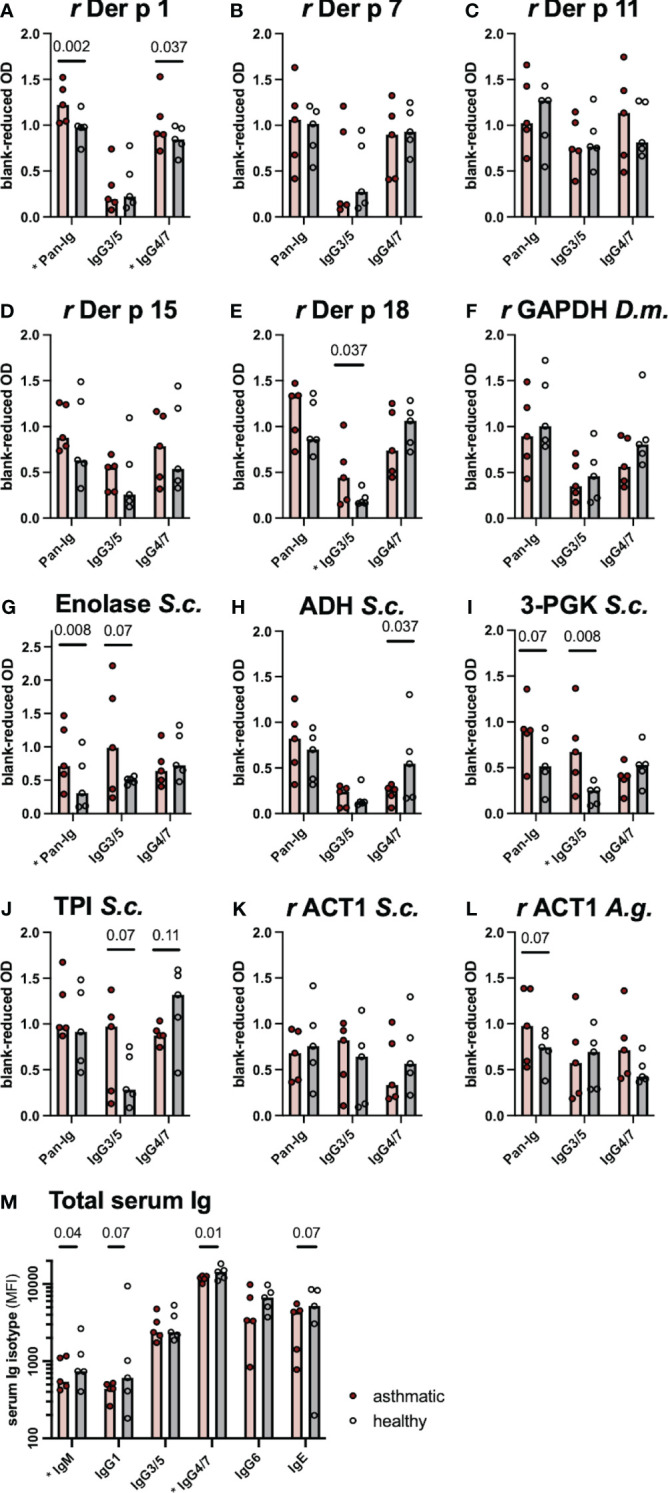
Differences in serum immunoglobulin (Ig) binding confirm antigen candidates. **(A–L)** Antigen-specific serum Ig binding was evaluated by ELISA with antigens from arthropods (*Der p Dermatophagoides pteronyssinus, D.m. Drosophila melanogaster*) or yeasts (*S.c. Saccharomyces cerevisiae, A.g. Absidia glauca*) coated on the plates. Ig binding was quantified for all serum Ig (Pan-Ig), IgG3/5, and IgG4/7. **(M)** Total Ig isotype concentrations were determined by bead-based assays. Ig was compared between asthmatic and healthy horses’ sera (n=5 per group). Group medians are indicated by bars and values of individual horses’ sera by dots. Group comparisons were performed by Wilcoxon signed-rank tests. P-values <0.15 are given and comparisons with significant differences (p<0.05) are indicated * at the respective x-axis label. *r* recombinant protein expressed in *E. coli*; GAPDH, glyceraldehyde-3-phosphate dehydrogenase 1; ADH, alcohol-dehydrogenase; 3-PGK, 3-phosphoglycerol-phosphokinase; TPI, triosephosphate isomerase; ACT1, actin; OD, Optical density; MFI, median fluorescence intensity.

Serum Ig binding to single proteins, identified as candidates by the immunoproteomics approach, was quantified by ELISAs coated with the respective single proteins. Differences of serum Ig binding to *r* Der p 1 (**Figure 2A**), *r* Der p 18 (**Figure 2E**) and the three yeast enzymes enolase (**Figure 2G**), alcohol dehydrogenase (ADH; **Figure 2H**), and phosphoglycerate kinase (3-PGK; **Figure 2I**) confirmed these as antigen candidates in SEA (**Figure 2**). Trends of Ig binding differences were observed for triosephosphate isomerase (TPI; **Figure 2J**) and actin (*r* ACT1 A.g.; **Figure 2L**) from yeasts (**Figure 2**; **Table 4**). Three antigen candidates tested, *r* Der p 7, *r* Der p 11, and *r* GAPDH *(Drosophila melanogaster)*, did not yield Ig binding differences and were not confirmed as likely relevant antigens in SEA (**Figures 2B, C, F**; **Table 4**).

If group differences were observed, Pan-Ig and IgG3/5 binding was higher with asthmatic horses’ sera, while IgG4/7 binding was usually higher with healthy horses’ sera matching the most common pattern on the 2D immunoblots (**Figure 1F**). Yet, IgG4/7 binding to *r* Der p 1 was also higher with asthmatic horses’ sera than with healthy horses’ sera (**Figure 2A**). Antigen-specific Pan-Ig binding was higher in asthmatics’ sera on *r* Der p 1 and yeast Enolase (both p<0.01, **Figures 2A, G**; **Table 4**) and tended to be higher on 3-PGK and actin (*r* ACT1) from *Absidia glauca* (both p=0.07; **Figures 2I, L**), but not actin (*r* ACT1) from *Saccharomyces cerevisiae* (**Figure 2K**). IgG3/5 binding was higher with asthmatic horses’ sera on *r* Der p 18 and 3-PGK from *Saccharomyces cerevisiae* (p<0.05, **Figures 2D, I**; **Table 4**) and also tended to be higher on Enolase and TPI (both p=0.07, **Figures 2G, J**; **Table 4**). IgG4/7 binding was higher with healthy horses’ compared to asthmatics’ sera on ADH (p=0.037; **Figure 2H**; **Table 4**) and tended to be higher on TPI (p=0.107; **Figure 2J** and **Table 4**).

The total serum Ig isotype concentrations (IgM, IgG1, IgG3/5, IgG4/7, IgG6, and IgE) were analyzed by bead-based assays to compare if differences in antigen binding are reflected in the total concentrations of IgG isotypes. Healthy horses’ sera had higher IgM and IgG4/7 concentrations compared to asthmatic horses’ sera (p<0.05; **Figure 2M**) and tended to contain more IgG1 and IgE (p=0.07; **Figure 2M**) even though the magnitudes of the differences were small. The total serum IgG3/5 concentrations were similar in healthy and asthmatic horses’ sera (**Figure 2M**). Thus, total concentration differences did not explain the higher IgG3/5 binding observed on several spots on immunoblots or to single antigens in ELISAs.

## 4 Discussion

As the relevant antigens in SEA have not been characterized comprehensively yet, we employed a bottom-up approach of immunoproteomics of *Der p* avoiding allergen selection bias in serological testing. Sera of environmentally matched healthy and severely asthmatic horses characterized by the established HOARSI (32) and clinical examination were compared. Our analysis did not yield significant matching effects of the environment (same barn and hay feeding) on the immunoproteomics results encouraging the possibility of also considering this approach if environmental matching is not possible.

Even though most protein spots yielded Ig binding indicating that the spots contain immunogenic proteins, the Ig binding patterns to all spots did not discriminate asthmatic from healthy horses well. The selection of relevant spots on 2D immunoblots was critical for the groups’ discrimination according to serum Ig binding. For these selected spots, a typical isotype pattern resulted. Sera from asthmatic horses yielded higher IgG3/5 binding for most protein spots, which was in part reflected in overall Ig binding (Pan-Ig). Asthmatics’ serum IgG4/7 binding was higher in a few spots of high MW but lower than healthy horses’ serum IgG4/7 binding in most low MW protein spots. The pattern of increased serum IgG3/5 but decreased IgG4/7 binding in SEA compared to healthy horses was furthermore observed for the antigen candidates confirmed by ELISA analyses. Differences between SEA and healthy horses’ sera in separate IgG isotypes were more pronounced than those in Pan-Ig, supporting the approach to analyze IgG isotypes separately. Particularly, for the antigens that yielded opposing IgG3/5 (higher in SEA) and IgG4/7 (higher in healthy) binding, this appears critical. Nevertheless, the ELISA analyses warrant further confirmation, for example, in more individuals.

Equine IgG3/5 responses are Th2-associated (25, 26) and have been described in humoral responses to Tetanus toxoid (40), helminths (42), and allergic responses in *Culicoides* hypersensitivity (17), while equine IgG4/7 is Th1-associated (25) and is induced in immune responses to virus infection and challenge (26). Accordingly, the pattern of increased Pan-Ig and IgG3/5 but decreased IgG4/7 binding to specific proteins observed here can indicate a systemic type-2 bias in SEA and a trend of excessive humoral responses. This could support an allergic pathogenesis of SEA as well as IgG-based mechanisms underlying the pathology. According to the general properties of equine IgG3 and IgG5, these mechanisms could be immune complex formation and Fcγ receptor binding to support respiratory burst and complement fixation (the latter by IgG3) (43). These pathological mechanisms have only been discussed for equine asthma (2, 19, 20). Similarly, mouse models of human allergic asthma point to the exacerbation of airway inflammation and hyperreactivity by allergen-specific IgG and immune complexes activating innate immune cells through FcγRs (21). However, induction of allergen-specific IgG4 is considered a key mechanism in allergen-specific immunotherapy in humans rendering IgG isotype differentiation important (21). In the present study, the binding of IgG4/7 from asthmatic horses’ sera exceeded that of healthy horses on a few spots (e.g., IDs 37, 151, 152, and 378) and Der p 1 in ELISAs. While it was not the common pattern here, occasional elevated IgG4/7 could indicate mixed responses, including Th1-associated mechanisms, after exposure to certain immunogens. Mixed, elevated T cell responses were indicated by increased IL-4, IFN-γ, and IL-17 expression of T helper cells of asthmatic horses after polyclonal stimulation of PBMC *in vitro* (44). Direct comparisons of antibody and cellular responses to specific antigens in SEA are, however, still pending, and cellular responses were not analyzed in the present study.

Nevertheless, total IgG4/7 concentrations were lower in asthmatic horses’ sera while IgG3/5 was similar between the groups. Accordingly, the overall production of the isotypes alone did not explain the differences in specific Ig binding observed and the particular binding patterns on different antigens need to be considered. Furthermore, total serum IgE concentrations were similar between the groups. This does not exclude type-2 pathogenesis of SEA as elevated total serum IgE is not a robust indicator of allergy in horses since it is impacted by many factors such as season, environment, and endoparasite burden (26). All horses in the present study were sampled in summer and fall and were matched by their environment with the same management including deworming (33), and the endoparasite burden of the horses included here was low. Accordingly, these factors with known impact on IgE were comparable for each matched pair of asthmatic and healthy horses.

Remarkably, the antigen candidates identified by immunoproteomics contained not only mite proteins but abundant fungal proteins, particularly those from yeasts like *Saccharomyces cerevisiae*. Most likely, yeast preparations were used in the culture of *Der p*, and thus also included in the proteins extracted from the whole culture and used for 2D immunoblots here. Nevertheless, yeasts are frequent contaminants in hay (45, 46) to which horses are exposed and yeasts are therefore a plausible antigen source in SEA epidemiology. *Candida albicans* (extract) was also a prioritized allergen in the analysis of serum IgE in equine asthma by a microarray (5). The herein-identified immunogenicity of yeast proteins and their potential relevance in SEA warrants additional caution interpreting results of other tests employing mite extracts and those using recombinant proteins produced in yeast, which could likewise contain yeast proteins. If the examined subjects’ skin reaction or Ig binding to the preparations is in parts based on yeast antigen binding, the resulting test interpretation for ‘mite reactivity’ can be severely biased.

The fungal proteins were highly immunogenic according to their overall Ig binding and could also be confirmed as antigen candidates in SEA according to specific serum Ig binding on ELISAs. Most corroborative were the identifications of Enolase, ADH, and PKI as antigens in SEA while further candidates such as GAPDH and HSP82 need to be investigated further.

Yeast Enolase 1 is an interesting antigen candidate as Enolase has been described as an allergen (Sac c Enolase), and cross-reactivity of Ig against yeast and mold enolase (Asp f 22, Pen c 22) is possible (47). Nonetheless, cross-reactivity between enolase and other allergens, like Asp f 6 from *Aspergillus fumigatus* and Hev b 6 from *Hevea brasiliensis* (latex) have been described (48), and Hev b 6 has also been considered an important allergen discriminating asthmatic (mild-moderate and SEA) and healthy horses’ IgE binding in a microarray screening (5). Accordingly, it is not entirely clear if yeast enolase is the antigen that has induced the Ig response in asthmatic horses in the present study, as SEA is only diagnosed when the condition is already chronic (1), and the initial immune response cannot be observed anymore.

The yeast enzyme ADH, which was confirmed as an antigen of SEA here, was also described as an aero-allergen in severe human asthma (Cand a 1 (49)). Fungal PGK identified here has not been described as an allergen but appears to be another new interesting antigen in SEA. Yeast TPI was only detected in spot 378 and yielded trends by ELISA supporting it as a potential antigen in SEA here, while its relevance might be clarified further. Other TPIs have been described as aero-allergens, such as Asp t 36, which was recently discovered as an allergen and is a member of the same protein class as Der p 25 (23, 50). The latter was, however, not identified as a prioritized antigen candidate here.

The mite antigen candidates identified in the present study contained mainly proteins described as allergens, which are immunogenic proteins capable of provoking adaptive Ig responses by definition. However, the immunoproteomics did not merely yield the major allergens described for *Der p* but a particular selection. This was also not just reflective of the *Der p* TP composition, emphasizing the need for relevant antigen identification beyond a source organism for meaningful analyses. If extracts are used, which contain only minor amounts of the relevant allergens or antigens as suggested by the comparison with *Der p* TP here, the results of serologic or skin testing with these will likely be inconclusive (23).

Der p 1, a major allergen in human house dust mite allergy (23, 51), was indicated most frequently by immunoproteomics and was confirmed as an antigen candidate in SEA by the following ELISA analysis with *r* Der p 1. However, Der p 7 and Der p 11 were not confirmed by IgG binding here even though Der p 7 was also among allergens discriminating asthmatic (mild-moderate and SEA) and healthy horses according to IgE binding on a microarray (5). As chitinase-3-like protein (group 15 allergen) identified by immunoproteomics was unavailable, Der p 18, a chitin-binding group 18 allergen, was tested for comparison and was also confirmed as an antigen in SEA here even though it was not highly prioritized by the criteria of the immunoproteomics analysis. Additionally, myosin, the two highly prioritized uncharacterized proteins, and mite proteins that were further identified as antigen candidates by immunoproteomics could be interesting future targets.

However, the present study was limited to serum Ig analyses, and despite the association of certain T helper cell responses and equine Ig isotypes in general (26), specific T cell responses to the antigens identified here remain to be confirmed and characterized. General T cell analyses resulted in conflicting indications of excessive type 2 but also type 1 and type 3 responses in SEA (44, 52). Therefore, it is possible that individual asthma predisposition, specific antigen exposure, and stage determine the polarization of adaptive immune responses contributing to SEA. Narrowing down the antigen candidates according to Ig responses can enable clarification of the antigens’ impact on SEA pathogenesis in further studies including Ig-based and T-cell-mediated mechanisms.

### 4.1 Conclusion

The bottom-up approach of antigen candidate identification by immunoproteomics using 2D immunoblots and IgG isotype binding analysis followed by LC-MS protein identification was successfully used to identify new mite (*Der p*) and yeast proteins as likely disease-relevant antigens in SEA. The identification of single yeast proteins as potential antigens in SEA etiology here opens a new perspective and also warrants additional caution in the selection of allergen preparations for testing in horses. Increased IgG3/5 binding to relevant antigens might be a characteristic feature of SEA. These results form the basis of more comprehensive serological analyses using defined separate proteins as antigens and specific IgG isotype binding detection. Such analyses can significantly contribute to a better understanding of SEA pathogenesis but warrant complementation by analyses of corresponding specific T-cell responses.

## Data availability statement

The data presented in the study are deposited in the ProteomeXchange repository, accession number PXD045568 (https://proteomecentral.proteomexchange.org/cgi/GetDataset?ID=PXD045568) or by Panorama Public (https://panoramaweb.org/Der-p-in-SEA.url).

## Ethics statement

The animal studies were approved by Veterinary Ethical Committees of all 26 cantons in Switzerland. The studies were conducted in accordance with the local legislation and institutional requirements. Written informed consent was obtained from the owners for the participation of their animals in this study.

## Author contributions

CS: Conceptualization, Data curation, Formal analysis, Funding acquisition, Investigation, Methodology, Project administration, Supervision, Validation, Visualization, Writing – original draft, Writing – review & editing. M-CJ: Data curation, Investigation, Methodology, Writing – review & editing. SL: Data curation, Investigation, Methodology, Writing – review & editing. SK-T: Investigation, Methodology, Resources, Writing – review & editing. VG: Writing – review & editing, Investigation, Methodology, Resources. SV: Methodology, Resources, Writing – review & editing. H-JH: Methodology, Resources, Writing – review & editing. CR: Methodology, Resources, Writing – review & editing. BW: Investigation, Methodology, Resources, Writing – review & editing. RH: Conceptualization, Funding acquisition, Methodology, Resources, Software, Writing – review & editing. DV: Conceptualization, Data curation, Formal analysis, Funding acquisition, Investigation, Methodology, Software, Validation, Writing – original draft, Writing – review & editing.

## Supplementary material

Supplementary Figure 1 Two-dimensional protein spot pattern. **(A)** A fused image of the protein spot pattern from 40 gels with tryptophan fluorescence is depicted with estimated pH ranges of the isoelectric points (IEP) and molecular weights (MW), **(B)** spot annotations with boundaries and Spot IDs, and **(C)** spots cut for proteomics analysis.

Supplementary Figure 2 Ig binding visualization of one group of spots. **(A)** Spot pattern in the gel (fused image, protein as tryptophan fluorescence); magnified area with Spots #06 – 51 (high MW, acidic); Serum Ig binding detection of 10 sera (left, red from asthmatic horses; right, dark blue from healthy horses) in side-by-side duplicates detected for **(B)** Pan-Ig, **(C)** IgG3/5, and **(D)** IgG4/7 binding. Images created with Delta 2D software.

Supplementary Figure 3 Immunodetection over all spots does not cluster asthmatic horses’ samples together nor environmentally matched pairs. Two-dimensional immunoblots of Der p TP incubated with serum samples from severely asthmatic or healthy horses **(A)**, immunodetection of Pan-Ig **(B)**, IgG3/5 **(C)**, and IgG4/7 **(D)** were quantified as normalized fluorescent intensity volumes (color coding of the heat maps) per each of the 440 spots detected. Using the Delta 2 D software, hierarchical clustering was performed (gene tree and sample tree, Euclidean Distance, complete linkage clustering) and visualized as heat maps with trees (cropped after 30 spots here). Duplicates of single sera are usually clustered together. Neither environmentally matched pairs (barns A–E) nor sera from asthmatic vs. healthy horses clustered systematically together.

Supplementary Figure 4 Ig isotype binding comparison per spot Ig binding for each of the spots of interest on 2D immunoblots is plotted as logarithmized fluorescence volume intensities of Pan-Ig (Cy3), IgG3/5 (Cy5), and IgG4/7 (Cy5). Bars represent means and SD. Per spot, each isotype binding was compared between asthmatic (red) and healthy (black) horses’ sera by 2-way ANOVA with Šidák’s multiple comparisons test. Comparisons with p<0.05 are indicated with asterisks.

Supplementary Table 1 Immunoproteomics results and Accession numbers.
