# Supplementary figures and images for: Immunoproteomics reveal increased serum IgG3/5 binding to Dermatophagoides and yeast protein antigens in severe equine asthma in a preliminary study
Source: Front Immunol. 2023 Dec 15;14:1293684. doi: 10.3389/fimmu.2023.1293684 (PMC10754955; doi:10.3389/fimmu.2023.1293684)

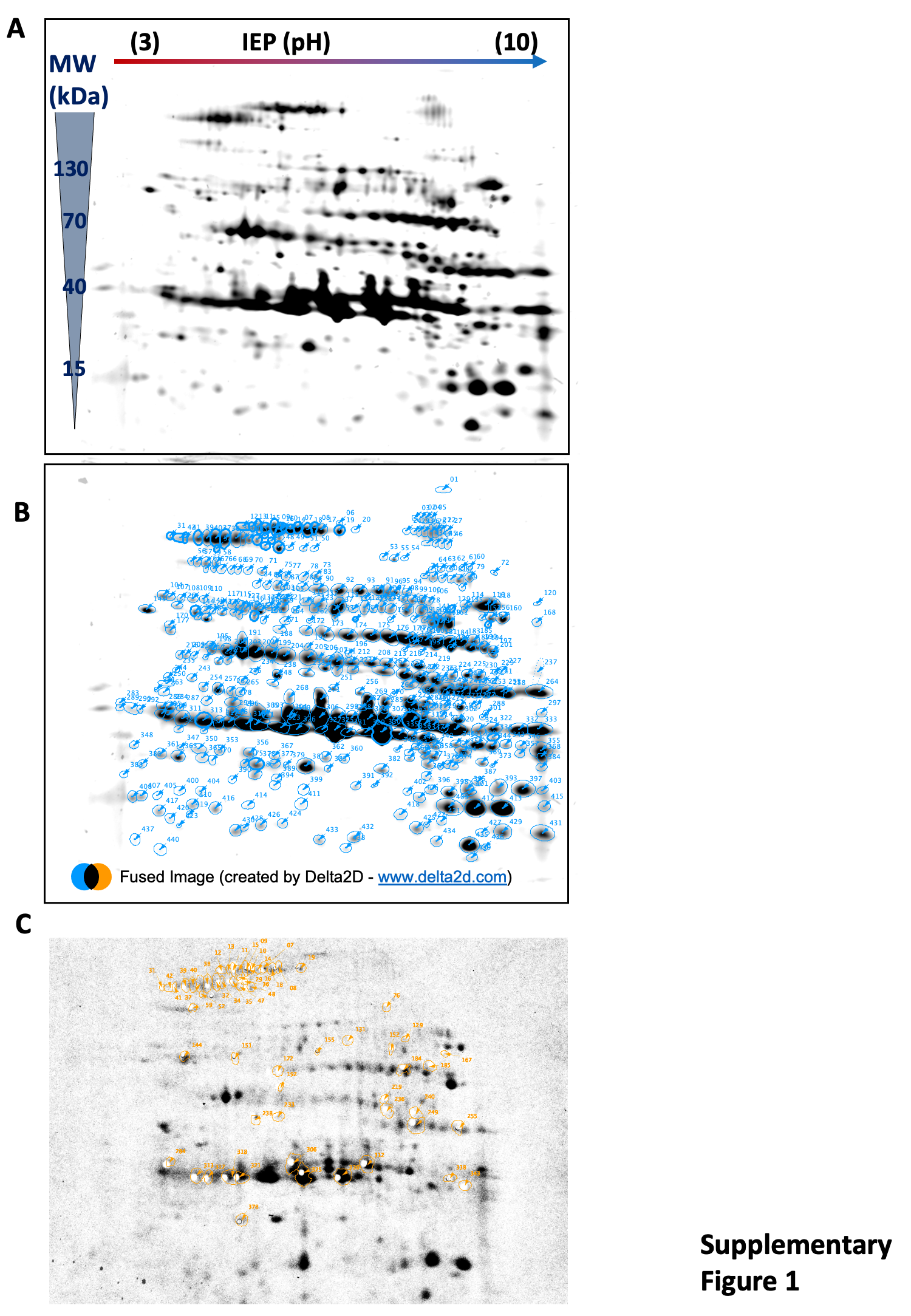

Supplement: Supplementary Figure 1 — Two-dimensional protein spot pattern. (A) A fused image of the protein spot pattern from 40 gels with tryptophan fluorescence is depicted with estimated pH ranges of the isoelectric points (IEP) and molecular weights (MW), (B) spot annotations with boundaries and Spot IDs, and (C) spots cut for proteomics analysis. [file Image_1.tiff]

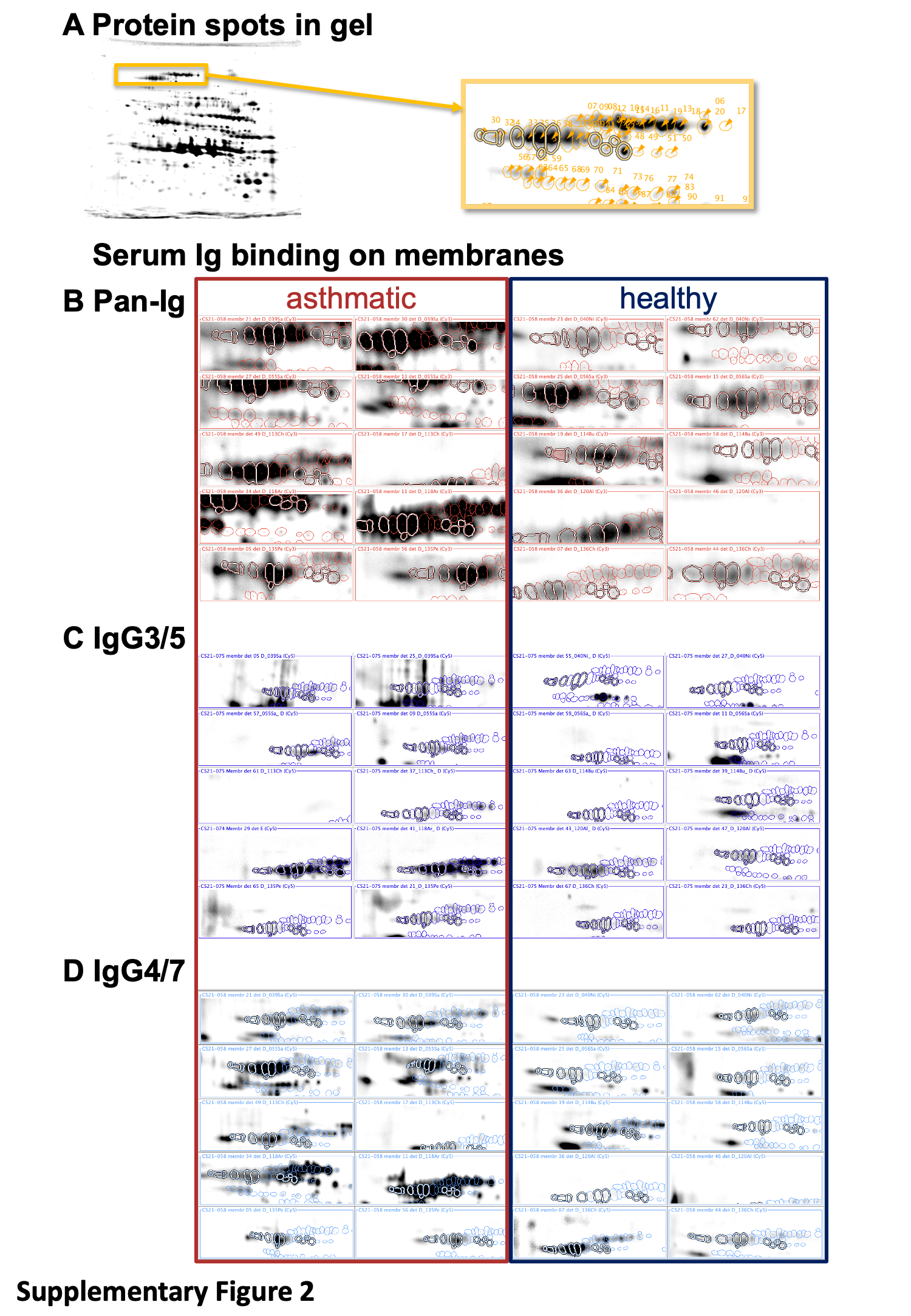

Supplement: Supplementary Figure 2 — Ig binding visualization of one group of spots. (A) Spot pattern in the gel (fused image, protein as tryptophan fluorescence); magnified area with Spots #06 – 51 (high MW, acidic); Serum Ig binding detection of 10 sera (left, red from asthmatic horses; right, dark blue from healthy horses) in side-by-side duplicates detected for (B) Pan-Ig, (C) IgG3/5, and (D) IgG4/7 binding. Images created with Delta 2D software. [file Image_2.tiff]

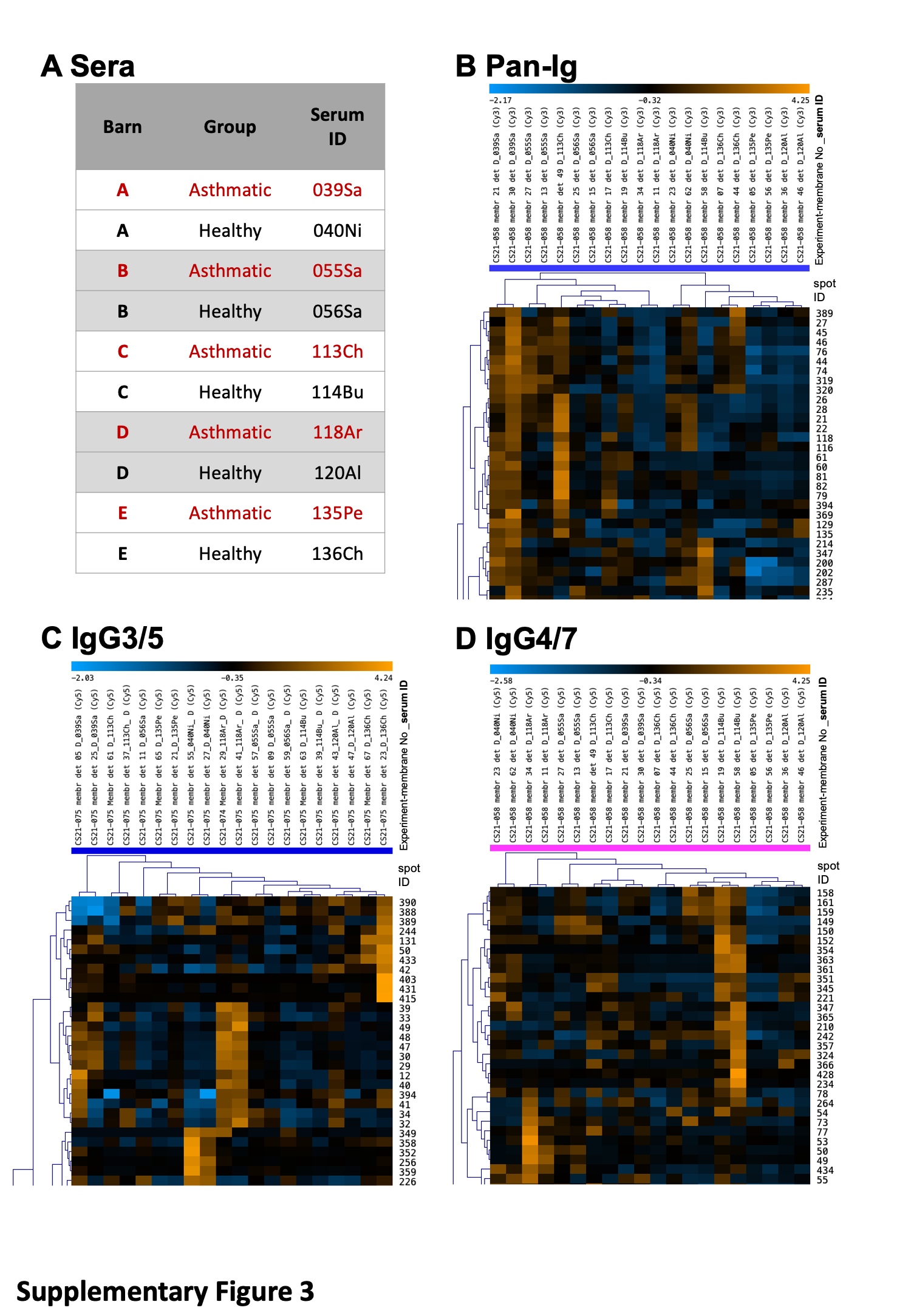

Supplement: Supplementary Figure 3 — Immunodetection over all spots does not cluster asthmatic horses’ samples together nor environmentally matched pairs. Two-dimensional immunoblots of Der p TP incubated with serum samples from severely asthmatic or healthy horses (A), immunodetection of Pan-Ig (B), IgG3/5 (C), and IgG4/7 (D) were quantified as normalized fluorescent intensity volumes (color coding of the heat maps) per each of the 440 spots detected. Using the Delta 2 D software, hierarchical clustering was performed (gene tree and sample tree, Euclidean Distance, complete linkage clustering) and visualized as heat maps with trees (cropped after 30 spots here). Duplicates of single sera are usually clustered together. Neither environmentally matched pairs (barns A–E) nor sera from asthmatic vs. healthy horses clustered systematically together. [file Image_3.tiff]

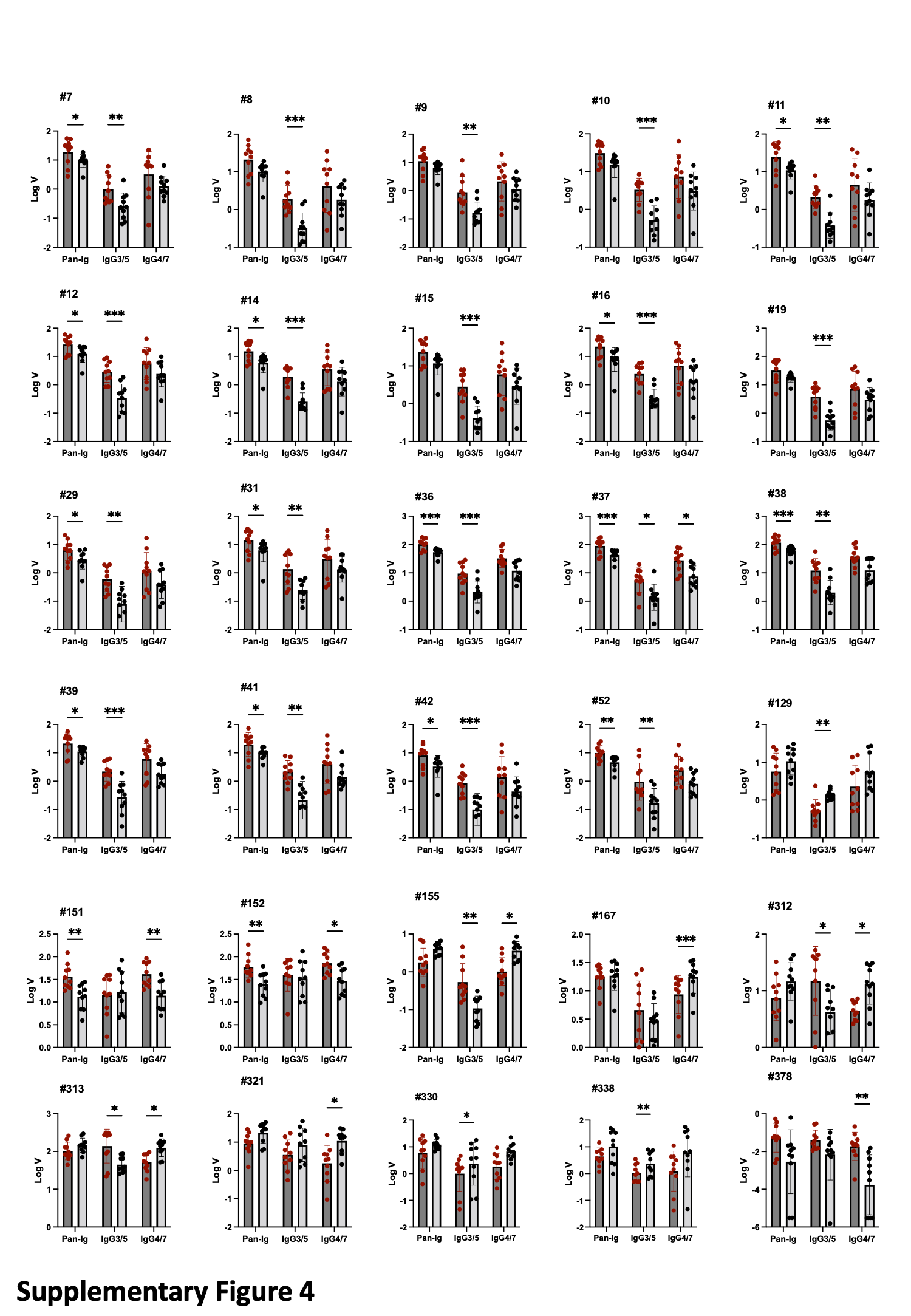

Supplement: Supplementary Figure 4 — Ig isotype binding comparison per spot Ig binding for each of the spots of interest on 2D immunoblots is plotted as logarithmized fluorescence volume intensities of Pan-Ig (Cy3), IgG3/5 (Cy5), and IgG4/7 (Cy5). Bars represent means and SD. Per spot, each isotype binding was compared between asthmatic (red) and healthy (black) horses’ sera by 2-way ANOVA with Šidák’s multiple comparisons test. Comparisons with p<0.05 are indicated with asterisks. [file Image_4.tiff]
